# Analysis of the Therapeutic Effect and Prognostic Factors of 126 Patients With Hypertensive Cerebral Hemorrhage Treated by Soft-Channel Minimally Invasive Puncture and Drainage

**DOI:** 10.3389/fsurg.2022.885580

**Published:** 2022-04-29

**Authors:** Jiaxun Wu, Sunfu Zhang

**Affiliations:** Department of Neurosurgery, The Third People's Hospital of Chengdu, Chengdu, China

**Keywords:** hypertensive cerebral hemorrhage, soft-channel minimally invasive puncture and drainage, efficacy, vasoactive factor, prognosis

## Abstract

**Background:**

Surgery is the main method for the clinical treatment of hypertensive cerebral hemorrhage. Traditional craniotomy faces the disadvantages of the long operation time, easy to cause secondary injury to patients during the operation, and prone to infection after the operation, which is not conducive to the rehabilitation of patients. At present, it is urgent to find a surgical scheme, which can clear hematoma in time, protect brain tissue, and effectively reduce surgical trauma in the clinic.

**Materials and Methods:**

The case database of our hospital was consulted, and the clinical data of patients with hypertensive intracerebral hemorrhage (HICH) treated with soft channel minimally invasive puncture and drainage from February 2018 to October 2021 were retrospectively analyzed. Patients were evaluated for efficacy, and the changes in serum C-reactive protein (CRP), tumor necrosis factor-α (TNF-α), interleukin-6 (IL-6), homocysteine (Hcy), endothelin (ET), and vasopressin (AVP) levels before surgery, 3 days after surgery, and 7 days after surgery were analyzed. Clinical data were collected and Logistic regression was used to analyze the prognostic factors.

**Results:**

Finally, according to the inclusion and exclusion criteria, 126 patients were selected as the research object. Among them, there were 24 cases (19.05%) of recovery, 47 cases (37.30%) of markedly effective, 34 cases (26.98%) of effective, 11 cases (8.73%) of ineffective, and 10 cases (7.94%) of death. The total effective rate was 83.33%. The hematoma was basically removed in 116 cases (92.06%). The average evacuation time of hematoma was (7.82 ± 1.63) days. Post-operative intracranial infection occurred in 2 cases (1.59%) and post-operative rebleeding occurred in 5 cases (3.97%). The average hospital stay was (34.16 ± 16.59) days. Serum CRP, TNF-α, IL-6, Hcy, ET, and AVP levels of all patients on the third and seventh days after surgery were lower than those before surgery, and those on the seventh day after surgery were lower than those on the third day after surgery (*p* < 0.05). The differences in pre-operative Glasgow Coma Scale (GCS) score, bleeding volume, ventricular rupture, complicated cerebral hernia, and attack time to surgery between the good prognosis group and the bad prognosis group were statistically significant (*p* < 0.05). Pre-operative GCS score, bleeding volume, ventricular rupture, complicated cerebral hernia, and onset time to surgery were all independent factors that affect the prognosis of patients (*p* < 0.05).

**Conclusion:**

Soft-channel minimally invasive puncture and drainage treatment of HICH has a significant effect, which is conducive to the complete removal of hematoma, reducing hospitalization time, while adjusting the balance and stability of various cytokines, and improving patient prognosis. Pre-operative GCS score, bleeding volume, rupture into the ventricle, complicated cerebral hernia, and time from onset to operation are all independent factors that affect the prognosis of patients.

## Introduction

Hypertensive intracerebral hemorrhage (HICH) is a common acute lesion of cerebral vessels. It is a space-occupying lesion, which is caused by rupture, and hemorrhage of the cerebral artery is caused by long-term high blood pressure in patients, which then compresses the surrounding brain tissue, with a high mortality rate (1). In the past, craniotomy was usually performed to remove the intracerebral hematoma in patients. However, craniotomy takes a long time, which easily causes secondary injury to patients and post-operative infection, which is not conducive to patients' recovery. In addition to effectively reduce the surgical trauma of patients, promptly removing the hematoma and protecting the brain tissue are of great significance for improving the efficacy of HICH and the prognosis of patients (2). Soft-channel minimally invasive puncture and drainage has the advantages of no need for general anesthesia, small wound, and shortened operation time. It can not only effectively reduce intracranial pressure but also avoid damage to brain tissue by positioning the intracranial hematoma through CT and guiding the minimally invasive puncture and drainage, so the prognosis of most patients is good (3). Studies have found that in the progression of HICH, the activation of inflammatory factors and the destruction of the balance of vasoactive factors in patients have an impact on hemodynamics and vascular permeability *in vivo* and will further aggravate the progression of intracranial hematoma and brain tissue damage. Therefore, monitoring the dynamic changes of inflammatory factors and vasoactive factors is conducive to judging the progression and prognosis of patients with HICH (4). At present, there are no innovative findings about the value of soft-channel minimally invasive puncture and drainage in the treatment of HICH, and there are also few reports on the risk factors that affect the efficacy of this treatment. In this study, the clinical data of patients with HICH who were treated with soft-channel minimally invasive puncture and drainage were retrospectively analyzed, the efficacy of this surgical scheme for HICH was explored, and the factors affecting the prognosis of HICH patients were analyzed.

## Materials and Methods

This study was approved by our Ethics Committee and all patients were informed and agreed. All the data have been confirmed.

The case database of our hospital was consulted, and the clinical data of patients with HICH who were treated with soft-channel minimally invasive puncture and drainage from February 2018 to October 2021 were retrospectively analyzed. Inclusion criteria were as follows: all the patients met the relevant diagnostic criteria for HICH in the Guidelines for Diagnosis and Treatment of Intracerebral Hemorrhage in China (5) and were not complicated with other types of cerebral hemorrhage confirmed by cranial CT; all patients were treated within 24 h of onset. There was no prior history of stroke and no complications of circulatory and respiratory diseases. All follow-up data were complete and true.

All patients received soft-channel minimally invasive puncture drainage. A 64-row spiral HEAD CT was used to stereotaxically locate the midpoint of the largest layer of intracerebral hematoma as the puncture site, and the puncture was performed at the temporal part to avoid the main middle meningeal artery. After local anesthesia, an incision of 0.6–0.8 cm was made on the scalp at the puncture site. A hole was made in the skull, and the dura was electrocoagulation under direct vision, followed by a postcruciform incision of the dura, and cerebral cortical vessels were avoided. A core 12F silica gel drainage tube was used to puncture the hematoma cavity. When old blood was educed, the drainage tube was implanted with 1.0 cm toward the hematoma, and a small amount of normal saline was slowly injected for washing. After the drainage tube was placed, the scalp was sutured for fixation, and the three-way tube and the special external drainage device were connected for drainage. During the operation, the drainage tube should pass through the center of the hematoma once as possible to avoid arbitrary adjustment of puncture direction. The strength should be controlled during aspiration, and fresh hemorrhage caused by violent aspiration of the hematoma under large negative pressure should not be allowed. After surgery, hematoma drainage was monitored dynamically under cranial CT, and urokinase was administered to dissolve the clot if necessary. After 3–5 days of catheter placement, the drainage tube was removed according to the drainage situation.

Patients were assessed according to the National Institute of Health Stroke Scale (NIHSS) score for efficacy that includes cured (no disability, 91–100% reduction in NIHSS score), markedly effective (achieving Grade 1–3 disability, 46–90% reduction in NIHSS score), effective (10–45% reduction in NIHSS score), and ineffective (<10% reduction or increase in NIHSS score). The total effective rate is the sum of recovery rate, apparent efficiency, and response rate. The death of patients in the two groups was recorded.

The clinical data that include the average evacuation time of hematoma, the incidence of post-operative intracranial infection, the incidence of post-operative rebleeding, and the average hospital stay were analyzed.

Serum C-reactive protein (CRP), tumor necrosis factor-α (TNF-α), interleukin-6 (IL-6), homocysteine (Hcy), endothelin (ET), and vasopressin (AVP) levels before surgery, 3 days after surgery, and 7 days after surgery in all patients were analyzed. CRP, IL-6, and TNF-α levels were detected by chemiluminescence enzyme analysis. The instrument adopts IMMULITE automatic chemiluminescence instrument produced by Siemens, and the detection reagent is provided by Siemens. The operation is carried out in accordance with the instrument regulations and reagent instructions. The levels of Hcy, ET, and AVP were determined by immunoturbidimetry.

All patients were followed up for 3 months. Patients with cured and effective clinical efficacy were divided into a good prognosis group, and patients with dead and ineffective were divided into a poor prognosis group. The basic conditions of all patients were recorded in detail, such as name, gender, age, admission, onset time, and follow-up for prognosis analysis.

SPSS22.0 software was used for processing. The continuous variable data of experimental data were expressed as mean standard deviation (SD) ($\bar{x}$ ± s) and adopted the t test. The classified variable data and descriptive analysis were expressed as (%) and adopted the χ^2^ test. Logistic regression was used to analyze the related factors that affect the prognosis of patients with HICH. The test level was α = 0.05, and *p* < 0.05 indicated that the difference was statistically significant.

## Results

Finally, according to the inclusion and exclusion criteria, 126 patients were selected as the research object. There were 77 men and 49 women, with an average age of 56.38 ± 9.84 years old and an average bleeding volume of 45.82 ± 6.51 ml. There were 88 cases of basal ganglia hemorrhage, 19 cases of lobar hemorrhage, 13 cases of thalamic hemorrhage, 6 cases of cerebellar hemorrhage, and 21 cases of cerebral hemorrhage breaking into the ventricle.

As shown in Table 1, there were 24 cases (19.05%) of recovery, 47 cases (37.30%) of markedly effective, 34 cases (26.98%) of effective, 11 cases (8.73%) of ineffective, and 10 cases (7.94%) of death. The total effective rate is 83.33%.

**Table 1:** Analysis of post-operative efficacy in all patients with hypertensive intracerebral hemorrhage (HICH) (*n*, %).

**Cured**	**Markedly effective**	**Effective**	**Ineffective**	**Die**	**Total effective rate**
24 (19.05%)	47 (37.30%)	34 (26.98%)	11 (8.73%)	10 (7.94%)	105 (83.33%)

As shown in Table 2, the hematoma was basically removed in 116 cases (92.06%). The average evacuation time of hematoma was 7.82 ± 1.63 days. Post-operative intracranial infection was occurred in 2 cases (1.59%) and post-operative rebleeding was occurred in 5 cases (3.97%). The average hospital stay was 34.16 ± 16.59 days.

**Table 2:** Improvement of post-operative clinical symptoms in all patients with hypertensive intracerebral hemorrhage (HICH) (%,_$\bar{x}$ ± s).

**Evacuation of hematoma (cases)**	**Evacuation time of hematoma (days)**	**Post-operative intracranial infection (cases)**	**Post-operative rebleeding (cases)**	**Average hospitalization time (days)**
116 (92.06%)	7.82 ± 1.63	2 (1.59%)	5 (3.97%)	34.16 ± 16.59

As shown in Table 3, the serum levels of CRP, TNF-α, IL-6, Hcy, ET, and AVP of all patients on the 3 and 7 days after surgery were lower than those before surgery, and those on post-operative 7 days were lower than those on post-operative 3 days, the differences were statistically significant (*p* < 0.05).

**Table 3:** Changes in serum inflammatory factors and vasoactive factors in all patients before and after treatment (*n*, $\bar{x}$ ± s).

**Time**	**CRP (mg/ml)**	**TNF-α (ng/ml)**	**IL-6 (pg/ml)**	**Hcy (μmol/L)**	**ET (ng/L)**	**AVP (ng/L)**
Pre-operative	30.58 ± 4.92	3.85 ± 0.68	264.18 ± 32.08	25.62 ± 3.74	94.16 ± 10.83	19.84 ± 8.73
3 days after operation	24.09 ± 4.35*	2.59 ± 0.41*	174.51 ± 22.46*	19.65 ± 2.95*	82.56 ± 9.84*	15.49 ± 6.18*
7 days after operation	14.92 ± 3.86^*#^	1.74 ± 0.34^*#^	108.63 ± 17.54^*#^	13.58 ± 2.44^*#^	74.16 ± 8.37^*#^	10.95 ± 3.27^*#^
*F*	4.865	3.259	5.016	3.568	4.152	4.328
*P*	0.008	0.025	0.006	0.021	0.015	0.012

Compared with that before surgery, *p < 0.05. Compared with the situation 3 days after surgery, ^#^p < 0.05.

As shown in Table 4, the differences in pre-operative GCS score, bleeding volume, ventricular rupture, complicated cerebral hernia, and attack time to surgery between the good prognosis group and the bad prognosis group were statistically significant (*p* < 0.05).

**Table 4:** Single-factor analysis of influencing prognosis of patients with hypertensive intracerebral hemorrhage (HICH) (*n*, %).

	**Good prognosis group (*n* = 105)**	**Bad prognosis group (*n* = 21)**	*****χ^2^*****	***P***
**Gender**				
Male	64 (60.95%)	13 (61.90%)	0.167	0.934
Female	41 (39.05%)	8 (38.10%)		
**Age**				
≥50 years old	68 (64.76%)	10 (47.62%)	2.181	0.139
<50 years old	37 (35.24%)	11 (52.38%)		
**Dyslipidemia**				
Yes	83 (79.05%)	17 (80.95%)	0.201	0.653
No	22 (20.95%)	4 (19.05%)		
**GCS score of pre-operative**				
≥8 points	85 (80.95%)	8 (38.10%)	5.824	0.007
<8 points	20 (19.05%)	13 (61.90%)		
**Amount of bleeding**				
≥50 ml	64 (60.95%)	18 (85.71%)	5.235	0.010
<50 ml	41 (39.05%)	3 (14.29%)		
**Bleeding site**				
Basal ganglia hemorrhage	76 (72.38%)	12 (57.14%)	2.241	0.524
Lobar hemorrhage	14 (13.34%)	5 (23.81%)		
Thalamic hemorrhage	10 (9.52%)	3 (14.29%)		
Cerebellar hemorrhage	5 (4.76%)	1 (4.76%)		
**Rupture into ventricle**				
Yes	7 (6.67%)	14 (66.67%)	5.983	0.006
No	98 (93.33%)	7 (33.33%)		
**Complicated with cerebral hernia**				
Yes	16 (32.65%)	9 (55.38%)	4.937	0.015
No	89 (67.35%)	11 (44.62%)		
**Time of operation**				
≥12 h	51 (48.57%)	8 (38.10%)	4.336	0.019
<12 h	54 (51.43%)	13 (61.90%)		

The prognosis of patients was taken as a dependent variable, and the factors with significant differences in Table 4 are taken as independent variables to be included in the Logistic regression model. The assignments of the dependent variable and independent variable are shown in Table 5.

**Table 5:** Assignment of independent risk factors affecting the prognosis of patients with hypertensive intracerebral hemorrhage (HICH).

**Variable**	**The assignment**
**Dependent variable**	
Prognosis	Good prognosis = 0, Bad prognosis = 1
**Independent variable**s	
GCS score of pre-operative	<8 points = 0, ≥8points = 1
Amount of bleeding	<50 ml = 0, ≥50 ml = 1
Rupture into ventricle	No = 0, Yes = 1
Complicated with cerebral hernia	No = 0, Yes = 1
Time of operation	<12 h = 0 ,≥12 h = 1

As shown in Table 6, pre-operative GCS score, bleeding volume, ventricular rupture, complicated cerebral hernia, and onset time to surgery were all independent factors that affect the prognosis of patients (*p* < 0.05).

**Table 6:** Multivariate analysis of prognosis of patients.

**Factor**	***B***	***SE***	***Walds***	**Sig**.	**Exp(B)**
GCS score of pre-operative	−2.438	0.286	4.695	0.021	2.152
Amount of bleeding	2.058	0.493	4.583	0.023	2.067
Rupture into ventricle	2.354	0.254	6.547	0.008	1.853
Complicated with cerebral hernia	2.743	0.395	5.413	0.011	1.587
Time of operation	2.038	0.216	3.159	0.037	1.957

## Discussion

Hypertensive intracerebral hemorrhage is a serious non-invasive cerebral hemorrhage disease (6). In patients with HICH, the intracranial artery rupture and hemorrhage and hematoma will solidify and accumulate to compress brain tissue, thereby resulting in intracranial hypertension and structural damage of brain tissue. Moreover, in severe cases, it will lead to cerebral hernia and neurological dysfunction (7). In the past, the treatment for patients with HICH was mainly evacuation of hematoma through craniotomy. Although the hematoma could be completely removed under the visual state, the craniotomy operation was complex and tedious, the operation took a long time, which was likely to cause re-injury to the patient's brain tissue and complicated infection after surgery, and the patients tolerated it. Therefore, it has become an important purpose to remove the hematoma and protect brain tissue in time, and at the same time, to reduce the surgical trauma of patients, so as to improve the surgical effect and the prognosis of patients (8, 9).

Soft-channel minimally invasive puncture and drainage, as a surgical approach with small trauma and low damage to patients, has been gradually applied to the clinical treatment of HICH in recent years (10). In this study, first of all, we used imaging methods, such as cranial CT, to locate the bleeding site, and at the same time, under the supervision of CT, drilled the skull and placed a special drainage tube to accurately puncture the hematoma, and slowly aspirated to remove the hematoma. Finally, urokinase is added to dissolve the massive hematoma and completely remove the residual hematoma (11). The results showed that the total effective rate and hematoma removal rate of 126 patients were 83.33 and 92.06%, respectively. The evacuation time and hospitalization time of hematoma were both relatively low. Only seven patients experienced post-operative intracranial infection and post-operative rebleeding. The reason was that when patients with HICH underwent soft-channel minimally invasive puncture drainage, the front end of the drainage tube was made of a material with good flexibility, so that secondary damage to brain tissue was not easy to occur during the intubation process. In addition, a closed drainage system was used during the operation, in which the drainage speed was manually controlled according to the changes in intracranial pressure, to prevent the occurrence of excessive drainage. While the occurrence of excessive drainage is prevented, the stimulation of the drainage hose on brain tissues is reduced, the removal efficiency of hematoma is ensured, and the operation time is shortened. Thereby being beneficial to improving the curative effect and improving the prognosis of patients (12).

Inflammation also plays a role in the progression of HICH. Studies have found that the activation of inflammatory factors in patients can affect hemodynamics, increase the permeability of the blood-brain barrier, and further aggravate the progression of intracranial hematoma (13). Serum CRP, TNF-α, and IL-6 are all sensitive inflammatory factors in the body. After the occurrence of HICH, they are affected by a severe immune response, and each index is secreted in a large amount in the body (14, 15). Hcy is related to the occurrence and development of cerebral hemorrhage. A high level of Hcy can damage vascular endothelium by promoting oxidative stress reaction in the body and destroying the balance of the coagulation system in the body, thus aggravating the symptoms of cerebral hemorrhage (16). ET and AVP both play important roles in regulating cardiovascular function and maintaining basic vascular tone function. When cerebral hemorrhage occurs, the balance of vasoactive factors, such as ET and AVP, is broken, and it is difficult to maintain the normal function of the blood vessels (17, 18). In this study, the examination time of inflammatory factors and vasoactive factors was 3 days and 7 days after surgery. After post-operative treatment, the abnormal inflammatory factors and vasoactive factors caused by surgical stress reactions have been improved. The results of this study showed that the levels of inflammatory factors and vasoactive factors were significantly reduced 3 and 7 days after surgery when compared with those before surgery. These results indicated that soft-channel minimally invasive puncture and drainage could effectively reduce intracranial pressure, regulate the balance and stability of various cytokines, improve the clinical symptoms patients with of HICH, and reduce brain tissue damage, which was of great significance for the prognosis of patients (19).

In this study, the clinical data of HICH patients with good prognosis and poor prognosis were analyzed and compared. The two groups had significant differences in pre-operative GCS score, bleeding volume, rupture into the ventricle, complicated with a cerebral hernia, and time from onset to operation (*p* < 0.05). Moreover, multivariate analysis showed that pre-operative GCS score, bleeding volume, rupture into the ventricle, complicated with a cerebral hernia, and onset time to surgery were all independent factors affecting the prognosis of patients (*p* < 0.05). Many studies have proved that the GCS score of patients with cerebrovascular diseases before surgery has a significant correlation with the prognosis. The higher the GCS score indicates that the lower the degree of brain damage is, the better the post-operative prognosis will be. It is a protective factor for patients with cerebral hemorrhage (20, 21). The larger the amount of cerebral hemorrhage, the more likely it is to form a huge and irregular hematoma, which will develop into a cerebral hernia in a short period of time, seriously affecting the circulation of cerebrospinal fluid, and compressing and damaging the brain tissue in different parts, thus leading to a poor prognosis. At the same time, the range of HICH breaking into the cerebral ventricle and whether to visit the doctor in time are also closely related to the prognosis (22). Therefore, pre-operative attention to the clinical intervention of patients with risk factors is of great value for improving the surgical efficacy and clinical prognosis of patients.

## Conclusion

Soft-channel minimally invasive puncture and drainage treatment of HICH has a significant effect, which is conducive to the complete removal of hematoma, reducing hospitalization time, while adjusting the balance and stability of various cytokines, and improving patient prognosis. Pre-operative GCS score, bleeding volume, rupture into the ventricle, complicated cerebral hernia, and time from onset to operation are all independent factors that affect the prognosis of patients.

## Data Availability Statement

The original contributions presented in the study are included in the article/supplementary material, further inquiries can be directed to the corresponding author/s.

## Ethics Statement

This study was approved by the Ethics Committee of our hospital (2018003). The patients/participants provided their written informed consent to participate in this study.

## Author Contributions

JW and SZ are mainly responsible for the writing of the article and guidance of the entire research. JW is mainly responsible for research design and data analysis. Both authors contributed to the article and approved the submitted version.

## Funding

This study was supported by Chengdu Science and Technology Bureau (ZX20210805420) and Sichuan Science and Technology Department (2021YFS0082).

## Conflict of Interest

The authors declare that the research was conducted in the absence of any commercial or financial relationships that could be construed as a potential conflict of interest.

## Publisher's Note

All claims expressed in this article are solely those of the authors and do not necessarily represent those of their affiliated organizations, or those of the publisher, the editors and the reviewers. Any product that may be evaluated in this article, or claim that may be made by its manufacturer, is not guaranteed or endorsed by the publisher.
